# Evolutionary History of the *Marchantia polymorpha Complex*

**DOI:** 10.3389/fpls.2020.00829

**Published:** 2020-06-26

**Authors:** Anna-Malin Linde, Weerachon Sawangproh, Nils Cronberg, Péter Szövényi, Ulf Lagercrantz

**Affiliations:** ^1^Plant Ecology and Evolution, Department of Ecology and Genetics, Uppsala University, Uppsala, Sweden; ^2^Biodiversity, Department of Biology, Lund University, Lund, Sweden; ^3^Division of Conservation Biology, School of Interdisciplinary Studies, Mahidol University, Kanchanaburi, Thailand; ^4^Institute of Evolutionary Biology and Environmental Studies, University of Zurich, Zurich, Switzerland

**Keywords:** *Marchantia polymorpha*, hybridization, bryophytes, incomplete lineage sorting, whole-genome sequencing, phylogeny, introgression

## Abstract

The potential role of introgression in evolution has gained increased interest in recent years. Although some fascinating examples have been reported, more information is needed to generalize the importance of hybridization and introgression for adaptive divergence. As limited data exist on haploid dominant species, we analyzed genomes of three subspecies of the liverwort *Marchantia polymorpha*. We used available genomic data for subsp. *ruderalis* and carried out whole-genome (PacBio) sequencing for one individual each of subsp. *montivagans* and subsp. *polymorpha* as well as Illumina resequencing of additional genomes for all three subspecies. The three subspecies were compared against *M. paleacea* as outgroup. Our analyses revealed separation of the three taxa, but all three possible topologies were richly represented across the genomes, and the underlying divergence order less obvious. This uncertainty could be the result of the divergence of the three subspecies close in time, or that introgression has been frequent since divergence. In particular, we found that pseudo-chromosome 2 in subsp. *montivagans* was much more diverged than other parts of the genomes. This could either be explained by specific capture of chromosome 2 from an unknown related species through hybridization or by conservation of chromosome 2 despite intermittent or ongoing introgression affecting more permeable parts of the genomes. A higher degree of chromosomal rearrangements on pseudo-chromosome 2 support the second hypothesis. Species tree analyses recovered an overall topology where subsp. *montivagans* diverged first and subsp. *ruderalis* and subsp. *polymorpha* appeared as sister lineages. Each subspecies was associated with its own chloroplast and mitochondrial haplotype group. Our data suggest introgression but refute a previous hypothesis that subsp. *ruderalis* is a new stabilized hybrid between the other two subspecies.

## Introduction

Hybridization among diverging lineages is not uncommon in nature, especially in rapidly radiating groups (Seehausen, 2004; Grant et al., 2005; Mallet, 2005, 2007; Fontaine et al., 2015). This process may attenuate divergence, introduce adaptive divergence from another population, or even create a new hybrid species. Even though hybridization is widespread, hybrid speciation is probably rare. When it happens, it is most often in the form of allopolyploid hybrid speciation (Soltis and Soltis, 2009) and documented instances of homoploid speciation are few (Rieseberg et al., 2003). A more frequent outcome of hybridization is introgression, the transfer of genetic material between species through hybridization and repeated backcrossing (Anderson and Hubricht, 1938). This may increase standing variation and adaptive divergence. Genetic recombination can quickly generate novel genotypes from existing nucleotide variation and may thus have an important role in adaptive evolution. Even if hybridization itself might be rare, introgression may provide new genetic variants at a higher frequency than *de novo* mutations (Ward and van Oosterhout, 2016; Martin and Jiggins, 2017). An increasing number of studies report evidence of introgression occurring across species boundaries as a consequence of hybridization (reviewed by Dowling and Secor, 1997) or horizontal gene transfer (Gogarten and Townsend, 2005; Galtier and Daubin, 2008) resulting in reticulate evolution with different parts of the genomes more or less exposed to gene transfer (Harrison and Larson, 2014).

Large scale genomic data provide an opportunity to characterize the history of hybridization and introgression (Payseur and Rieseberg, 2016). A complication for the analysis of such data is that incongruence between gene trees and the species tree could arise not only from hybridization/introgression but also from incomplete lineage sorting (ILS). In ILS ancestral polymorphisms persist over speciation events followed by chance fixation or persistent polymorphism in descendant lineages. Large population sizes and closely timed speciation events will increase the frequency of incongruent gene trees arising from ILS, and make the speciation and hybridization history more difficult to reveal (Rieseberg et al., 1999; Mossel and Roch, 2010).

Most genomic studies of hybridization and introgression have so far been conducted on organisms with a diploid dominant generation. It is thus of interest to study more cases having a dominant haploid generation and a short-lived sporophyte generation. In bryophytes, the diploid sporophyte is the actual hybrid combining the parental genomes (comparable to the F1 generation in a vascular plant). Meiosis takes place in the spore capsule, prior to spore formation, so the haploid spores represent recombinants of the parental genomes (comparable to the F2 generation in vascular plant). The spores are usually produced in massive amounts and wind-dispersed. After spore germination, the gametophyte phenotype is directly exposed to selection, no variation is masked by dominant alleles, so that favorable genes transferred to a new genomic background can potentially show a fast penetration in populations through clonal growth or secondary back-crossing (Shaw, 1994, 1998; Natcheva and Cronberg, 2007; reviewed by Natcheva and Cronberg, 2004).

*Marchantia polymorpha* L. is often treated as a complex of three subspecies which together has a cosmopolitan distribution, although introduced in some parts of the Southern Hemisphere (Paton, 1999). *Marchantia polymorpha* is one of the most studied species of liverworts but aspects of its phylogenetic relationships remain poorly resolved (Nishiyama et al., 2004; Qiu et al., 2006; Wickett et al., 2014), even after completion of whole-genome sequencing which was published in 2017 (Bowman et al., 2017). It has been used in botanical research for centuries, but has now been revived as a modern model plants to understand plant genetics and evolutionary processes (Shimamura, 2016). It is a thalloid liverwort, which can reproduce both sexually and asexually. Bryophytes (liverworts, mosses and hornworts) are the oldest of the extant lineages of land plants, and their position in the plant tree-of-life makes them interesting for studies concerning the evolution of land plants.

**FIGURE 1:**
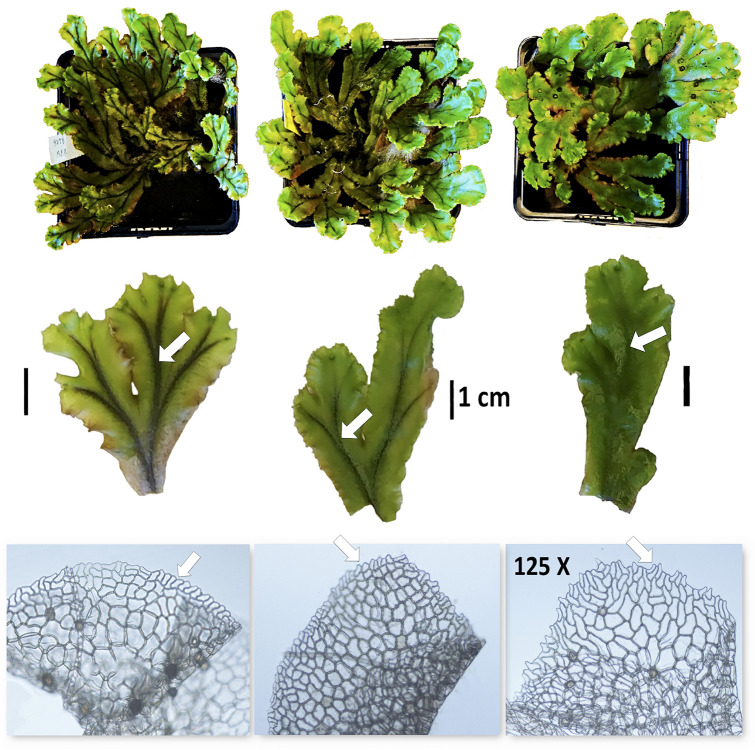
Images of *M. polymorpha* subsp. *polymorpha* **(left)**, *M. polymorpha* subsp. *ruderalis* **(middle)** and *M. polymorpha* subsp. *montivagans* **(right)**. Note the differences in the size of thalli (first row), the thickness of the black midrib of thalli (second row, arrow) and morphology of innermost ventral scales with 125X magnification (third row, arrow).

Following an early morphological taxonomic delimitation by Nees (1838) the *M. polymorpha-*complex was subdivided into three independent species. This subdivision was formalized by Burgeff (1943) as *M. polymorpha, M. alpestris* (Nees) Burgeff, and *M. aquatica* (Nees) Burgeff. Burgeff based this subdivision on restricted interfertility between the taxa in a reciprocal crossing experiment. The crosses between female *M. alpestris* and male *M. aquatica* rendered a relatively high frequency of viable spores (50–70% in all of five attempts with different accessions), whereas the other combinations were completely sterile (including the reciprocal cross, female *aquatica* x male *alpestris*) or nearly so. He was also able to repeatedly backcross female recombinants from the *alpestris* x *aquatica* cross with male *M. aquatica*. Later, observation of a recombinant in isozyme electrophoresis was taken as evidence of the occurrence of gene exchange between taxa (Bischler-Causse and Boisselier-Dubayle, 1991). Accordingly, the three taxa were instead recognized at the intraspecific level with the names commonly accepted today, subsp. *polymorpha*, subsp. *ruderalis* Bischl. & Boissel.-Dub. and subsp. *montivagans* Bischl. & Boissel.-Dub. Due to a typification error (the Linnean type for *M. polymorpha*, turned out to be the taxon found in aquatic environment), subsp. *ruderalis* refers to *M. polymorpha*, subsp. *montivagans* refers to *M. alpestris* and subsp. *polymorpha* refers to *M. aquatica, sensu* Burgeff. We use the names at subspecies level throughout this study, but we return to the question about taxonomic ranking in the discussion. The three subspecies of *M. polymorpha* are morphologically differentiated. *M. polymorpha* subsp. *polymorpha* shows thalli with distinct black continuous dark median line and appendage on innermost ventral scales with entire margin, whereas subsp. *montivagans* shows thalli without dark median line and appendage on innermost ventral scales with dentate margin. *M. polymorpha* subsp. *ruderalis* shows intermediate morphology between the other two subspecies by having thalli with discontinuous median line and appendage on innermost ventral scales with crenulated (projecting as low to sharp teeth) margin as shown in Figure 1 (Paton, 1999; Atherton et al., 2010). The subspecies have been estimated to have diverged in the Late Miocene (ca. 5–7 MYA) (Villarreal et al., 2016). They are ecologically and partially geographically separated but can sometimes be found sympatrically (Schuster, 1992, reviewed by Shimamura, 2016) suggesting opportunities for hybridization. Among the three subspecies, subsp. *ruderalis* is the most common and it has been proposed to have originated as a relatively new stabilized hybrid between the two other subspecies, adapted to disturbed man-made habitats (Schuster, 1983, 1992). However, a limited electrophoretic study using four isozymes gave no support for this hypothesis (Boisselier-Dubayle and Bischler-Causse, 1989).

## Materials and Methods

### Plant Material and DNA Extraction

**FIGURE 2:**
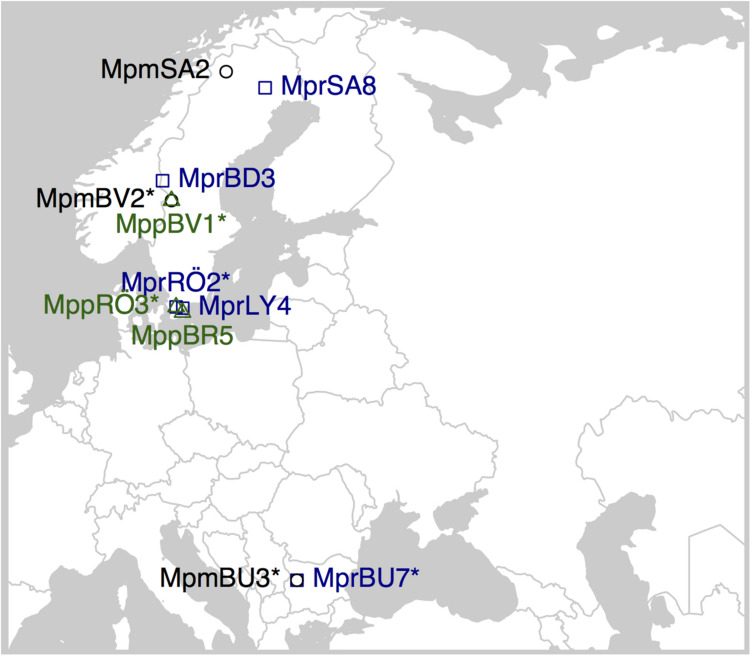
Sampling locations for the *M. polymorpha* specimens used in this study. Overlapping symbols have an asterisk in the sample ID when sampled at the same location. MPM, *Marchantia polymorpha* subsp. *montivagans*; MPP, *M. polymorpha* subsp. *polymorpha*; MPR, *M. polymorpha* subsp. *ruderalis*.

We sequenced 11 individuals of *M. polymorpha*; five representing subsp. *ruderalis*, three representing subsp. *polymorpha*, and three representing subsp. *montivagans*. All individuals were collected from locations in Sweden and Bulgaria (Supplementary Table 1 and Figure 2). All living samples we used for DNA extraction were kept in culture at the Department of Biology (Lund University). DNA extraction was performed with Qiagen DNeasy Plant Minikit using young thallus tissues for Illumina sequencing, and with a modified CTAB protocol (Healey et al., 2014) for PacBio sequencing. The R package “ggmap” (Kahle and Wickham, 2013) was used to create Figure 2.

### Genome Sequencing and Genome Assembly

One individual each of *M. polymorpha montivagans* (sample id MpmSA2) and *M. polymorpha polymorpha* (sample id MppBR5) were sequenced with Single-molecule real-time (SMRT) sequencing technology developed by Pacific BioSciences on a PacBio Sequel System with Sequel chemistry and sequence depth of 50X (Roberts et al., 2013). The reads were assembled using HGAP (Chin et al., 2013) and assembly statistics was assessed using QUAST (Gurevich et al., 2013) version 4.5.4, BUSCO (Simão et al., 2015) version 3.0.2 and CEGMA (Parra et al., 2007) version 2.5. BUSCO was used with the “Eukaryota odb9” dataset. Assembly quality and completeness are summarized in Supplementary Table 2.

For *M. polymorpha ruderalis* the publically available reference genome draft v.3.1 (Bowman et al., 2017) together with a chromosome-scale genome assembly (Diop et al., 2019) was used. The rest of the individuals in Supplementary Table 1 were sequenced using Illumina HiSeq X sequencing platform with pair-end reads of 2 × 150 bp. The reads were mapped against the three *M. polymorpha* genome assemblies as described below. The genome of *M. paleacea* subsp. *diptera* was sequenced and assembled as described in details in Radhakrishnan (2017) and very short as following: Short-insert pair-end libraries were produced using the NEBNext Ultra DNA Library Prep Kit (New England Biolabs, Ipswich, MA, United States) and long-insert mate-pair libraries were produced using Nextera Mate-Pair DNA library prep kit (Illumina, San Diego, CA, United States), following the manufacturer’s protocol. The reads were trimmed using Trimmomatic v0.33 (Bolger et al., 2014) and SPAdes (Bankevich et al., 2012) were used to assemble the contigs. Scaffolding of the contigs was performed using the scaffolder of SOAPdenovo (Luo et al., 2012).

### Preparation of Data: Alignment of Genomic Fragments (GFs)

The four genome assemblies (*M. polymorpha ruderalis, M. polymorpha montivagans, M. polymorpha polymorpha* and *M. paleacea diptera* were aligned using progressiveCactus (Paten et al., 2011a, b) version 2016-11-30 with default settings and with the following topology [*paleacea*, (*ruderalis*, *montivagans*, *polymorpha*)]. Cactus has been designed specifically to output HAL (hierarchical alignment) (Hickey et al., 2013). The resulting HAL-file was converted to MAF format using hal2maf from the HALtools utility (Hickey et al., 2013). The MAF-file was filtered with MafFilter (Dutheil et al., 2014) to extract the genomic fragment (GF) alignments. Only blocks where sequences from all four species occurred exactly once were kept. Additional filtering steps were carried out to match lengths and gaps. The GFs were concatenated (within scaffold borders) and fragmented into approximately 20,000 nt pieces. In total there were 2861 GFs of a length of approximately 20,000 nucleotides each and a total length of 60 MB, which corresponds to c. 25% of the total genome size. In order to assess effects of different GF lengths, shorter or longer GFs were tested, which gave the same results.

Sequences for the additional 2–5 short read-sequenced individuals from each subspecies were added to the GFs as follows. The four taxa alignments were split to generate a reference sequence set for each taxon. Illumina reads from nine additional genotypes were processed with BBDuk^1^ to trim and filter reads. The resulting reads were mapped to their respective subspecies reference set with BBMap (see text footnote 1). Generated bam-files were then used to generate a vcf file with freebayes (Garrison and Marth, 2012), followed by BCFtools consensus (Narasimhan et al., 2016) to produce a consensus sequence for each genotype. These consensus sequences were then added to the original four taxa alignments and realigned with FSA (Bradley et al., 2009).

### RNA Extraction and Genome Annotation

RNA was extracted from *M. polymorpha polymorpha* and *M. polymorpha montivagans* at two different time points with different light conditions, lightness and darkness. The RNA extraction was performed using RNeasy Plant Mini kit (Qiagen). The sequenced RNA raw data were assembled in two ways: a *de novo* assembly using Trinity v.2.4.0 (Grabherr et al., 2011) and genome-guided assembly using a combination of Hisat2 v.2.1.0 (Kim et al., 2015) and StringTie v.1.3.4 (Pertea et al., 2015). In the latter case, the raw reads were first trimmed using Trimmomatic v.0.36 (Bolger et al., 2014). SamTools v.1.8 (Li et al., 2009) and Gffread, belonging to the Cufflinks v.2.2.1 package (Trapnell et al., 2013) were used for intermediate file sorting and format conversion steps. The final file formats for the *de novo* and the genome-guided assemblies are FASTA and GFF3, respectively. The two libraries (sampled in light/darkness) per sample were assembled separately.

Genome annotation was done using Maker version 3.01.2-beta (Holt and Yandell, 2011) in two runs. The NBIS annotation toolkit^2^ was used for some of processing steps. In the first run the options *est2genome* and *protein2genome* were set to one, to obtain a first set of genes used to train the *ab initio* tools. The transcriptome assemblies were entered as *est*, respectively, *est_gff*. Swissprot (downloaded 2018-10-31 from https://www.uniprot.org/downloads) and the published proteome of *M. polymorpha ruderalis* were given as protein support. Augustus v. 3.2.3 (Stanke and Morgenstern, 2005) were trained using a non-redundant and AEDfiltered (≥ 0.3) set of proteins without isoforms from the first Maker run. GeneMark-ES were trained using gmes_petap.pl – ES –training with the protein2genome Maker output file as – evidence. In the second run of Maker, *est2genome* and *protein2genome* were set to 0, and the parameter files from the training of the two *ab initio* tools were entered. For both runs, “*always_complete*” was set to 1, and *alt_splice* and *run_evm* were set to 0. Species-specific repeat libraries identified (described below) were entered as *rmlib*. BUSCO v. 2.5 (Simão et al., 2015) with the “Eukaryota odb9” dataset were used to check the completeness of transcriptomes and annotations. For *M. paleacea* the transcriptomes of *M. polymorpha* subspecies were used as *est* in the first data set, giving a smaller set of conserved genes used to train the *ab initio* tools. In the second round they were given as *alt_est.* Only scaffolds larger than 10,000 were considered. The transfer of gene models from the references of each subspecies to the additional samples was also done using Maker but with the reference transcriptome used as *est* and with *est2genome, always_complete* and the hidden option *est_forward* set to 1.

### Preparation of Data: Alignment of Coding Sequences

The clustering of orthologs was done using OrthoVenn (Wang et al., 2015) based on the predicted output file from Maker, one file per species, with default settings (*e*-value cutoff: 10^–5^, inflation value: 1.5) and 9957 single-copy gene clusters were extracted for further phylogenetic analyses. The orthologous proteins were aligned using MUSCLE (Edgar, 2004) and the coding sequences (CDSs) were aligned based on the protein alignments using trimAL (Capella-Gutiérrez et al., 2009) with the backtranslate option to keep the information about codon positions. Only CDSs longer than 300 bases were kept. For the concatenation of CDSs the *concat* command of the SeqKit (Shen et al., 2016) tool was used.

### Repeat Annotation

RepeatModeler (Smit and Hubley, 2008-2015) (version 1.0.8_RM4.0.7) was used for *de novo* repeat family identification. The output was used as repeat library for RepeatMasker version 4.0.7 (Smit et al., 2013-2015).

### Phylogenetic Analysis: Mitochondria and Chloroplast DNA

The organellar DNA sequences were treated as single loci. Three different phylogenetic methods were used i.e., MrBayes, neighbor joining (NJ), and RaxML methods. MrBayes version 3.2.6 (Huelsenbeck and Ronquist, 2001; Ronquist and Huelsenbeck, 2003; Ronquist et al., 2012) was used to reconstruct the Bayesian phylogenetic tree using the best fitting substitution model of sequence evolution, selected using Modelgenerator version 85.1 (Keane et al., 2006). For chloroplast DNA this was GTR + G (lset nst = 6 rates = gamma) and for mitochondrial DNA GTR + G + I (lset nst = 6 rates = invgamma). Bootstrapped neighbor joining trees were also reconstructed using the “nj” and “boot.plylo” functions in the R package ape v. 5.2 (Paradis and Schliep, 2019). In addition, phylogenetic trees were reconstructed using RAxML with the nucleotide substitution model “–m GTRGAMMA” and 100 bootstraps using fast bootstrap (-x).

### Phylogenetic Analysis: Genomic Fragments (GFs) and Transcripts

Individual ML-trees were reconstructed for all GF and transcript alignments using the maximum likelihood method based RAxML version 8.2.4 (Stamatakis, 2014). The nucleotide substitution model chosen for all trees was “–m GTRGAMMA”. The GTR model of nucleotide substitution was chosen for all trees as it is the most general model, performing well for most real-world sequence data (RAxML manual), and 100 bootstraps using fast bootstrap (−x). The same methods were used with the concatenated versions of GFs and transcripts where all alignments for each type of data were joined together to one large alignment.

ASTRAL version 5.6.2 (Mirarab et al., 2014; Mirarab and Warnow, 2015) was used to estimate a species tree from the multiple GF/transcript trees. It takes a set of unrooted RAxML trees as input and gives as output an unrooted species tree, which is the tree that agrees with the largest number of quartet trees induced from the input tree set. It can handle ILS and is often more accurate than the concatenation method, except when the level of ILS is low (Mirarab et al., 2014; Mirarab and Warnow, 2015). If the bootstrap replicates for each alignment is included, ASTRAL performs a multi-locus bootstrapping. 100 bootstrapped replicates were done.

As a complement, to evaluate consistency, we also applied a bayesian approach using MrBayes for constructing phylogenetic trees and BUCKy for analyzing the complete set of trees. For MrBayes the Perl script “mb.pl” from the TICR pipeline^3^ was used with default settings. This output was used as input for BUCKy version 1.4.4 (Larget et al., 2010; Mirarab et al., 2014) to estimate the dominant history of sampled individuals and how much of the genome that supports each relationship based on Bayesian concordance analysis. These concordance factors are given with a credibility interval taking into account the uncertainty in gene tree estimates. We first used the default prior (1) and then tested a second prior (0.01), with the same results.

### Identification of Synteny and Other Chromosome-Level Comparisons

Chromosemble from the Satsuma2 packages (Grabherr et al., 2010) was used to order and orient the scaffolds of the *MpmSA2 and MprBR5* assemblies to 8 pseudo-chromosomes according to synteny with the *M. polymorpha ruderalis* genome. Only scaffolds larger than 100,000 were included. Satsuma2 package comprising SatsumaSynteny2, BlockDisplaySatsuma and MicroSyntenyPlot, was used to identify synteny matches, collect this information into synteny blocks, and visualize synteny as dotplots.

Gene order conservation was calculated using MCScanX_h (Wang et al., 2012) on the orthologous transcript data set. A collinear pair was defined as one orthologous pair lying directly adjacent to another orthologous pair, in both genomes compared and two or more adjacent genes are needed to be called a collinear block (-s 2 -m 0). Due to the fragmented nature of the genomes of subsp. *montivagans* and subsp. *polymorpha* the values are underestimations. The R package PopGenome (Pfeifer et al., 2014) was used to calculate pairwise nucleotide diversity between subspecies (d_*xy*_).

### Introgression Analyses

As a means to distinguish between ILS and introgression, we applied three variants of the ABBA-BABA test – Pattersons D statistic (Durand et al., 2011), Martins f statistic (Martin et al., 2013) and Bd-fraction (Pfeifer and Kapan, 2017) – using the R package “PopGenome” (Pfeifer et al., 2014). Local ancestry inference was conducted using Loter (Dias-Alves et al., 2018), with default settings and three ancestral populations (subsp. *ruderalis*, subsp. *polymorpha* and subsp. *montivagans*). When analyzing MpmBU3 or MppBV1 those individuals were excluded from their respective ancestral populations.

## Results

### Phylogenetic Inference

**FIGURE 3:**
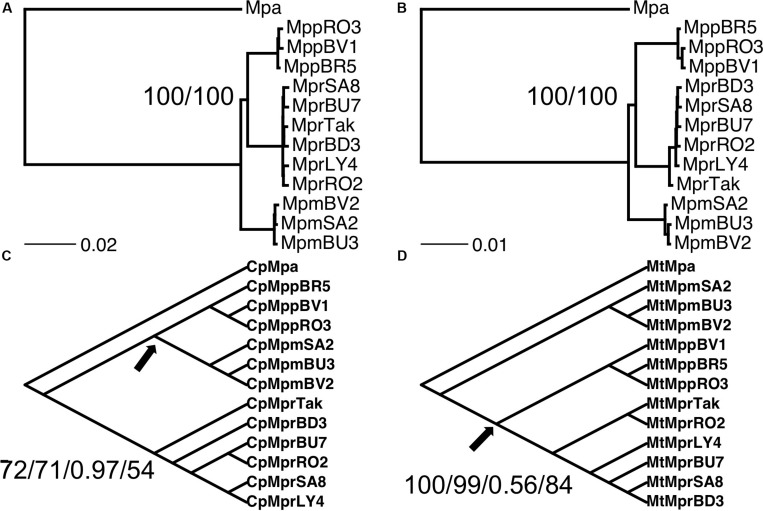
Phylogenetic relationship between the three subspecies of *M. polymorpha.* **(A)** Phylogenetic tree obtained from a concatenated alignment of genomic fragments (GFs) with branch length corresponding to substitutions per site. The first node support value corresponds to the RAxML bootstrap value for the concatenated alignments. Included also, as a second support value, is the bootstrap value from the ASTRAL species tree analysis. **(B)** Phylogenetic tree obtained from a concatenated alignment of coding transcripts, with branch length corresponding to substitutions per site. Node support values corresponding to RAxML bootstrap value for concatenated alignments/bootstrap value from ASTRAL species tree analysis. **(C)** Phylogenetic tree obtained from the chloroplast genome, with RAxML bootstrap/NJ bootstrap/MrBayes support value/RAxML bootstrap values using only genes. **(D)** Phylogenetic tree obtained from the mitochondria genome, with RAxML bootstrap/NJ bootstrap/MrBayes support value/RAxML bootstrap values using only genes. For clarity, due to the very low number of substitutions, the trees in **(C,D)** are cladograms and the branches are not proportional to substitutions per site. The relationship within each subspecies clade might vary between the trees produced with the different methods. Node support values are always shown for the node where two of the subspecies cluster together, while support values were always high for the first two splits. Note also that the chloroplast and mitochondria genomes consistently congruent with the nuclear genome subspecies designation, no transfer of chloroplasts or mitochondria between supspecies is detected in this data set.

In total, the genomes of six individuals of *M. polymorpha* subsp. *ruderalis*, three individuals of *M. polymorpha* subsp. *polymorpha* and three individuals of *M. polymorpha* subsp. *montivagans* are included in this study together with the genome of one individual of *M. paleacea* subsp. *diptera* used as outgroup. Only autosomes were included. Phylogenetic reconstructions in general displayed a clear separation of the three subspecies. However, the branching order of the three taxa was less obvious. Analyses based on complete nuclear DNA (irrespective of data set and phylogenetic method used) placed subsp. *polymorpha* and subsp. *ruderalis* as sister species. Subsp. *montivagans* is placed as sister lineage to subsp. *polymorpha* + subsp. *ruderalis* (Figures 3A,B). Using complete chloroplast DNA, subsp. *ruderalis* diverged first with subsp. *montivagans* and subsp. *polymorpha* as sister taxa (Figure 3C). Even though the support for these branching orders varied depending on tree reconstruction method, the length of the defining branch was always short. For complete mitochondrial DNA, the branching order was the same as that of the nuclear data, but with varying support and extremely short branches (Figure 3D).

These phylogenetic patterns could be the results of the divergence of all the three subspecies within a short period of time. This could lead to unresolved gene trees, or more than one supported topology due to ILS. Alternatively, recent hybridization might have obscured a previously clear branching order. To differentiate between these scenarios, we calculated concordance factors (CFs) for the three possible branching orders of the three subspecies. If, as previously suggested, subsp. *ruderalis* arose through a recent hybridization event between the other two subspecies we would expect gene trees clustering subsp. *ruderalis* and *polymorpha* (topology 1), and those clustering *ruderalis* and *montivagans* (topology 2), but not those grouping *montivagans* with *polymorpha* (topology 3).

**FIGURE 4:**
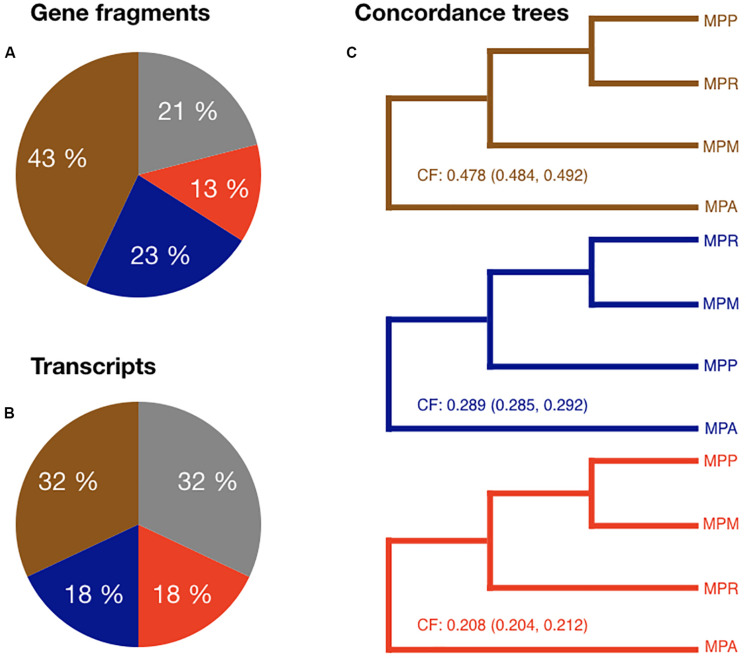
**(A)** Pie chart showing the proportion of each topology among the gene fragment RAxML trees. Gray represent trees with a bootstrap value <70. **(B)** Pie chart showing the proportion of each topology among the transcript RAxML trees. Gray represent trees with a bootstrap value <70. **(C)** Brown tree: Primary concordance tree. Node values are the concordance factors (CFs) including confidence interval for CFs. Blue and red trees: Minor concordance trees. Node values are the concordance factors including confidence interval for CFs.

The most abundant topology was the one favored in the nuclear species trees, topology 1 comprising 32/43% of all gene/GF trees and a CF of 0.49, followed by topology 2 (18/23% of all trees and CF = 0.28) (Figure 4). Even though topology 3 was the least abundant one, it constituted a considerable fraction of all supported individual trees (13/18%) and a CF of 0.21. In these analyses, trees with a bootstrap support of less than 70 were considered to be non-significant (21% for GFs and 32% for transcripts). These data contradict the proposed recent hybrid origin of subsp. *ruderalis*. Rather, the high frequency of supported trees for all three possible topologies suggests a similar age of the three subspecies and frequent ILS, possibly accompanied by more ancient hybridization and introgression.

**FIGURE 5:**
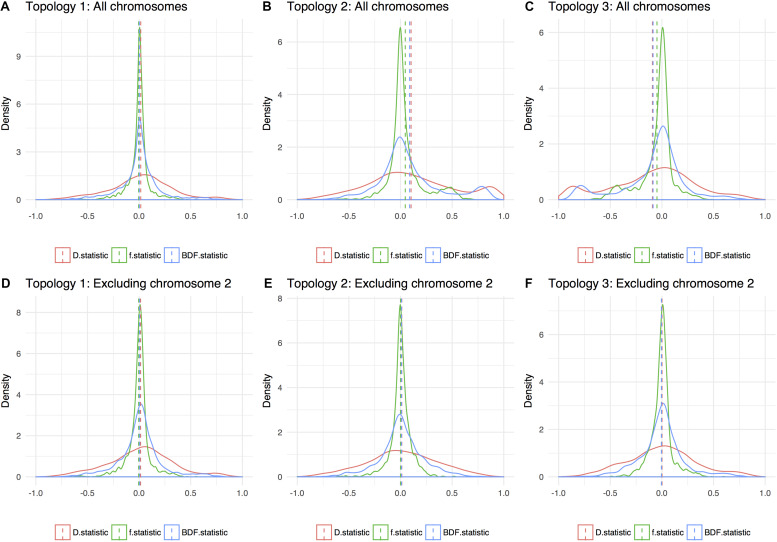
Density plot of three variants of introgression statistics (ABBA-BABA tests): D statistics, f statistics, and BDF statistics. Dashed lines is the genome average for the different topologies and they are shown with **(A–C)** and without chromosome 2 **(D–F)**, respectively. Excluding chromosome 2, all three possible topologies give an averaged value of around 0, indicating a star tree. A value that deviates from 0 indicate hybridization or that the wrong phylogenetic tree topology is given.

In ABBA-BABA tests, an excess of ABBA sites over BABA sites indicative of introgression is signaled by a significant positive deviation from zero (see e.g., Heliconius Genome Consortium, 2012). Assuming a phylogeny according to the obtained species trees for nuclear data, all calculated statistics were close to zero and non-significant (see Figures 5A–C). This result support the conclusion that ILS is prevalent, and that subsp. *ruderalis* is of similar age as the other two subspecies, and not a recent hybrid between the two.

### One Chromosome Has Experienced a Distinct Phylogenetic History

**FIGURE 6:**
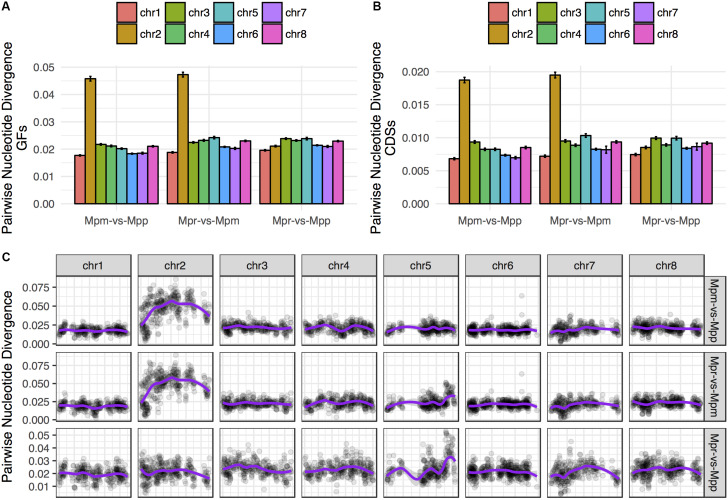
The alignments including subsp. *montivagans* chromosome 2 show increased nucleotide divergence. **(A)** Pairwise nucleotide divergence averaged over all GFs, separated by chromosome. **(B)** Pairwise nucleotide divergence averaged over the orthologous transcripts data set, separated by chromosome. **(C)** Chromosome scans of pairwise divergence show that the increased divergence covers most of chromosome 2 and that there is a larger spread of values.

The genome-wide pattern seen in the phylogenetic analysis is not representative for all chromosomes. For the trees based on data representing chromosome 2 in subsp. *ruderalis* the concordance factors (CFs) for the primary concordance tree are 0.906 and 0.930 for GFs and transcripts, respectively. This is in contrast to all other chromosomes where CFs are less than 0.5. In agreement with this pattern, GF and transcript alignments corresponding to subsp. *ruderalis* chromosome 2 showed a strikingly higher nucleotide divergence between subsp. *montivagans* and both the other subspecies (Figures 6A,B). Thus, all comparisons including subsp. *montivagans* and alignments comprising sequences from subsp. *ruderalis* chromosome 2 showed a divergence at least twice as high as any other comparison not including chromosome 2 and subsp. *montivagans*. The higher divergence is seen over a large part of the chromosome except at one end where it is at a comparable level to the rest of the genome (Figure 6C).

We might expect alignments corresponding to subsp. *ruderalis* chromosome 2 to have a large effect on phylogenetic reconstructions and the resulting species tree. To evaluate this, data were reanalyzed excluding alignments representing subsp. *ruderalis* chromosome 2. The analyses still support the same species tree with subsp. *polymorpha* together with subsp. *ruderalis* as sister to subsp. *montivagans*, but the branch lengths are now even shorter. We also calculated introgression-statistics (ABBA-BABA tests) for all three possible topologies. Excluding chromosome 2, all measures of introgression are non-significant and close to zero for all three possible topologies (Figures 5D–F). Thus, for analyses based on all chromosomes except number 2, there is no evidence of a deviation from a strict bifurcating evolutionary history. Rather, the analyses support an almost star like tree topology and frequent ILS.

**FIGURE 7:**
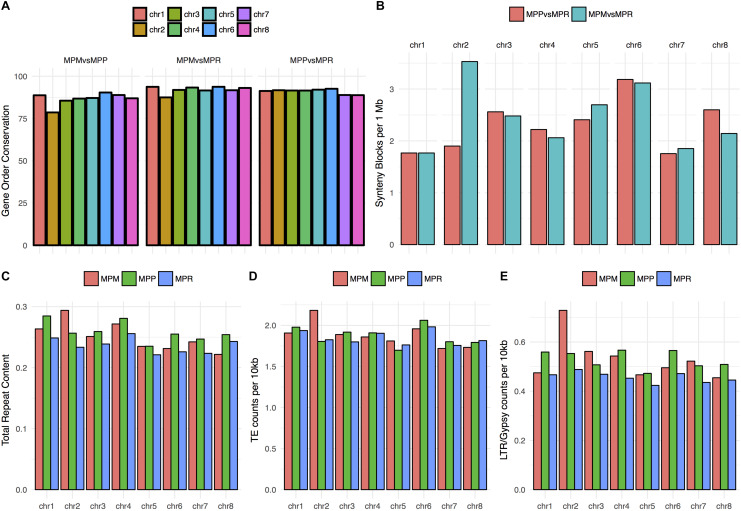
**(A)** Percentage of gene order conservation in pairwise comparisons, using subsp. *ruderalis* chromosome coordinates as reference. The gene order conservation is significantly lower for chromosome 2 in the two comparisons including subsp. *montivagans* (χ2 = 37.033; *P* = 1.162e-09 resp. χ2 = 11.708; *P* = 0.0006224). **(B)** Number of synteny blocks per 1 Mbases for the comparisons between subsp.*ruderalis* chromosomes and subsp. *polymorpha* pseudochromosomes (red) and between subsp. *ruderalis* chromosomes and subsp. *montivagans* pseudochromosomes (cyan). A higher number of synteny blocks correlates with a higher number of synteny breaks and more rearrangements. The corresponding synteny dot plots are shown in Supplementary Figures 1, 2. **(C)** Total repeat content per chromosome, represented as total repeat length per chromosome/pseudochromosome length. **(D)** Counts of transposable elements (Simple repeats, low complexity repeats and unknown are excluded) per 10,000 bases. **(E)** Counts per 10,000 bases of a specific transposable element, LTR/Gypsy, which is overrepresented in subsp. *montivagans* pseudochromosome 2.

As a means to better understand the distinct patterns observed for chromosome 2, we searched for other aspects of these sequences where they might deviate from the general pattern. The level of gene order conservation, as measured with MCscanX, comparison including both subsp. *montivagans* and chromosome 2, was significantly lower than other comparison (MPM vs MPP: χ^2^ = 37.033; *P* = 1.162e-09, MPM vs MPR: χ^2^ = 11.708; *P* = 6.224e-04) (Figure 7A). Even though assembly contiguity of the three subspecies differs, comparisons between chromosomes within subspecies are still valid.

Dot plots on chromosome-based alignments of genomic fragments also reveal more rearrangements for comparisons including chromosome 2 and subsp. *montivagans* (Supplementary Figures 1, 2; also visualized in Figure 7B). Furthermore, subsp. *montivagans* chromosome 2 sequences have a higher repeat content than those corresponding to other chromosomes. No such inflated content was observed for chromosome 2 sequences in the corresponding analysis of subsp. *ruderalis* and subsp. *polymorpha* (Figures 7C–E).

### One Chromosome Shows Evidence of Recent Introgression

In an effort to detect more recent introgression events, all individuals from the three subspecies were investigated separately. At a genome-wide scale, no clear evidence of introgression was detected but analyzing 2861 GFs, 53 RAxML trees showed a phylogenetic topology where single individuals clustered with the wrong subspecies. For chromosome 1 approximately 8% of the input individual GF RAxML trees (23 out of 323 GFs) gave a topology indicating introgression between one individual and another subspecies. The corresponding values for the other chromosomes were lower and varied between 0 and 3.4%. Quartet CFs were calculated for the 54 possible quartet combinations (three individuals of subsp. *montivagans* x three individuals of subsp. *polymorpha* x six individuals of subsp. *ruderalis* × one individual of *Marchantia paleacea*). The CFs are expected to be independent on quartet combination in the absence of occasional introgression events in one of a few individuals. Analyzing chromosome 1 separately, two samples deviated from this expected pattern, MppBV1 and MpmBU3. In quartets including MppBV1 the CFs were higher than expected for the topology [(MPP, MPM), MPR] and while the CFs for quartets including MpmBU3 were higher than expected for [(MPR, MPM), MPP]. These data may indicate introgression between subsp. *polymorpha* and *montivagans* in the first case, and between subsp. *montivagans* and *ruderalis* in the second case.

**FIGURE 8:**
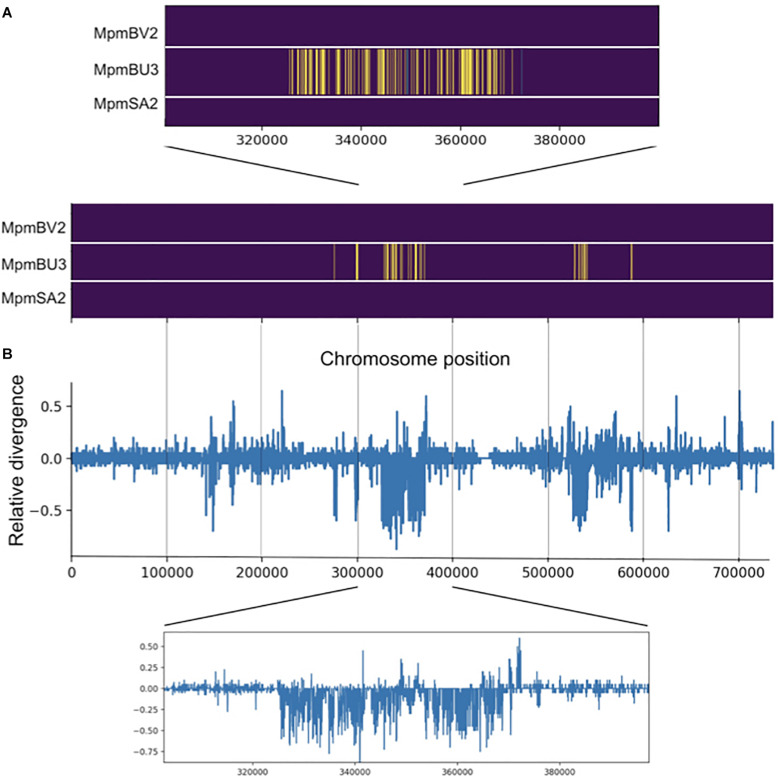
Evidence for introgression is observed for one subsp. *montivagans* individual. **(A)** Local ancestry inference for three subsp. *montivagans* individuals derived from Loter software. Image plot of pseudo-chromosome 1 illustrating ancestral origin, subsp. *montivagans* (purple), *ruderalis* (yellow) and *polymorpha* (green). The *x*-axis shows SNP number along chromosome 1. **(B)** Plot of difference in genetic divergence between on the one hand MpmBU3 versus subsp. *ruderalis* individuals, and on the other hand MpmBU3 versus other subsp. *montivagans* individuals (d_*MpmBU*__3____*_*ruderalis*_* – d_*MpmBU*__3____*_*montivagans*_*), i.e., positive bars show genes that are diverged between MpmBU3 versus subsp. *ruderalis* (but not within subsp. *montivagans*), whereas negative bars reveal genes that differ to other accessions of subsp. *montivagans* (but not with subsp. *ruderalis*). Insets show magnifications of the data along the pseudo-chromosome.

To further explore possible introgression in individuals MppBV1 and MpmBU3, we performed local ancestry inference using the software Loter (Dias-Alves et al., 2018). Evidence of introgression was observed in both individuals on chromosome 1 (Figure 8 and Supplementary Figure 3). For MpmBU3, one major area at the center of chromosome 1 and a few smaller regions were inferred as having subsp. *ruderalis* origin (Figure 8A). The major area is separated into smaller regions separated by tracts of subsp. *montivagans* origin, suggesting that several generations with recombination have occurred.

An alternative interpretation to recent introgression for the inferred tracts of “foreign” origin could be ILS that for some reason is concentrated to a few areas on chromosome 1. An expectation from a recent introgression event in MpmBU3 is that introgressed tracts should be more similar to corresponding regions of subsp. *ruderalis* than to those of its own subsp. *montivagans*. This expectation does not hold for an ILS scenario. A plot of relative divergence along chromosome 1 (Figure 8B) clearly shows that in MpmBU3, areas with a concentration of tracts inferred as originating from subsp. *ruderalis*, show a lower divergence to pure *ruderalis*, than to the other two *montivagans* individuals (without evidence of introgression). Thus, our data support that introgression has occurred in BU3 some generations ago.

Similarly, for MppBV1 local ancestral inference identified one region on chromosome 1, with inferred ancestry from subsp. *montivagans* and one smaller region with inferred ancestry from subsp. *ruderalis* (Supplementary Figure 3). Again, the areas with a concentration of tracts inferred as subsp. *montivagans* show a lower divergence to pure *montivagans*, as compared to the pure *polymorpha* individuals. Thus, we find evidence for introgression resulting from hybridization a limited number of generations ago in a couple of individuals, but we find no evidence for any older fixed introgression events.

## Discussion

This paper reports the first large-scale phylogenomic analysis of the taxonomically controversial *M. polymorpha* complex in which three taxa of uncertain phylogenetic relationship have variably been treated at subspecies or species levels. The general phylogenetic pattern observed comprise three distinct taxa that diverged close in time. In line with this, the data also suggest frequent ILS resulting in a high degree of inconsistent gene trees. Still, species tree analyses recovered an overall topology where *M. polymorpha montivagans* diverged first and *M. polymorpha ruderalis* and *M. polymorpha polymorpha* appeared as sister species.

Our data thus refute the hypothesis proposed by Burgeff (1943) and Schuster (1983, 1992) that subsp. *ruderalis* is a homoploid hybrid, formed by hybridization between subsp. *montivagans* and *polymorpha*. This hypothesis has been questioned by Boisselier-Dubayle et al. (1995) based on a limited data set, but our study is the first to test this hypothesis at the level of whole genomes.

In addition to the general phylogenetic patterns, our more detailed analyses revealed a more complex pattern with evidence suggesting hybridization and introgression between subspecies. One unexpected finding was that pseudo-chromosome 2 in subsp. *montivagans* showed several aberrant features. Most of this chromosome displayed more than twice the amount of genetic divergence to both subsp. *polymorpha* and *ruderalis*, as compared to other chromosomes. This increased divergence for chromosome 2 was also accompanied by higher degree of chromosomal rearrangements. Two scenarios that could explain this pattern include (1) hybridization with an unknown closely related species, and (2) extensive hybridization between *M. polymorpha* subspecies. For both scenarios additional factors must be included to explain that the effect is confined to a single chromosome. The first scenario requires genomic mixing with an unknown closely related species in the past or present. It also requires that one chromosome has been more resistant to elimination of foreign chromosomal material through repeated backcrossing to subsp. *montivagans*. The second scenario implies that hybridization between subspecies has been frequent in the past, and that a single chromosome has been more resistant to this hybridization. None of these scenarios seems very likely, but our analysis of potential hybridization in single individuals might favor the second scenario over the first. This is comparable with whole-genome studies of malaria parasite vectors belonging to the *Anopheles gambiae* complex (Fontaine et al., 2015). The mosquito species belonging to this complex show pervasive autosomal introgression, so that only a small part of the genome, mainly on the X-chromosome, has not crossed the species boundaries. The branching order determined from the X-chromosome was used to construct the true phylogeny, and then this topology was used to trace back the major introgression events. The authors concluded that their proposed historical branching order was represented by only 1.9% of 50-kb windows across the entire genome, but when this topology was recovered, the divergence times were consistently more distant, relative to the alternative topologies. With a similar scenario, the divergence of the aberrant pseudo-chromosome 2 in subsp. *montivagans* may represent the true phylogenetic separation from the other two subspecies.

For two individuals in our limited sample, MpmBU3 and MppBV1, we saw evidence of introgression with subsp. *ruderalis*, respectively, subsp. *montivagans* in restricted parts of their genomes. In both cases, we registered that the parental species pairs occurred in sympatry at the collection sites. For these pairs, Burgeff (1943) recorded low spore germination rate (9%) when crossing male subsp. *montivagans* and female subsp. *ruderalis* but considerably higher spore germination rate (50–70%) in the cross between female subsp. *montivagans* and male subsp. *polymorpha*. The reciprocal crosses did not render any viable spores. Burgeff also demonstrated that backcrossing with males of subsp. *polymorpha* was possible with progeny from the latter, more successful cross, which gives some experimental support for our suggestion that several generations with recombination may have occurred with regard to MpmBU3 and MppBV1.

In our cases, we registered that both the parental species pairs occurred in sympatry, and from our total population sampling it seems that sympatric populations are more common than generally recognized in the literature. For example, Damsholt (2002) states that the taxa rarely meet but mentions two exceptional sites in Scandinavia where subsp. *montivagans* and subsp. *ruderalis* (Sädvajaure, Pite Lappmark, Sweden), respectively, subsp. *montivagans* and subsp. *polymorpha* (hot springs by Landmannalaugar, Iceland) occur together. Only in the latter place, some intermediate plants possibly suggested introgression in that unusual habitat. If we were to generalize these observations, hybridization between subspecies may well have been frequent, but in most cases foreign DNA fragments are rapidly shortened through backcrossing and recombination. This could lead to pattern similar to the one obtained through frequent ILS and could thus fit with the observation of an almost star-like phylogeny of the three subspecies. Subspecies *montivagans* has a montane distribution, whereas both *polymorpha* and *ruderalis* occur in lowland areas, the former typically in wetlands and the latter in drier and more ruderal contexts including places subject to forest fire. It is therefore not unlikely that climatic oscillations during the Pleistocene has periodically brought the subspecies in closer contact than they normally are today and allowed for more frequent hybridization, so that the pattern we see may be a product of historic sympatry.

The haploid-dominant lifecycle might explain a spatially limited genetic exchange after hybridization. Interspecific hybridization in bryophytes results in diploid hybrid sporophytes formed after fertilization. The hybrid sporophyte is physically connected to the female plant and short-lived. The two parental genomes can recombine during meiosis, which takes place in the sporophyte, to form spores. The true hybrid is the sporophyte and the spores produced in the sporophyte are recombinants that have a mix of genes from both parents and can be referred to as hybrid segregates comparable to the F_2_ generation of angiosperms with hybrid origin. Spores are formed in tetrads and each tetrad is the result of a single meiotic event. The sporophytes produce not less than 300,000 spores in *M. polymorpha* (O’Hanlon, 1926) and thus 75,000 meiotic events are likely to take place during production of recombinant spores. If extensive amounts of genomic admixture in recombinants lead to incompatibilities, and therefore spore abortion or hampered spore germination, we can expect surviving individuals to show a strongly asymmetric representation of the parental genomes. A similar explanation has been suggested for high mortality and limited admixture observed in hybrid F_2_ progeny in peat mosses (Natcheva and Cronberg, 2007). In sympatric populations of the pleurocarpous mosses *Homalothecium lutescens and sericeum*, mildly admixed individuals were relatively common and strongly admixed individuals were sometimes seen in both gametophyte and sporophyte generations (Sawangproh, 2019, Sawangproh et al., in press). This suggests that the admixed alleles were transmitted between generations and that sympatric populations behave as true hybrid zones.

Pseudo-chromosome 2 in subsp. *montivagans* did not only show higher nucleotide divergence and more chromosomal rearrangements. Is also showed a higher proportion of transposons. At present it is premature to speculate about a causal relationship between these observations, but it is possible that more rearrangements discriminating subsp. *montivagans* pseudo-chromosome 2 from its homologs attenuate chromosome pairing of this chromosome in hybrids. If so, such attenuated pairing and thus reduced introgression could explain why subsp. *montivagans* pseudo-chromosome 2 is still more diverged.

Our sampling is restricted to a limited part of the whole distribution range, and differentiation patterns could possibly deviate in other regions. However, it is worth to notice that the nuclear, mitochondrial and plastid genomes of the geographically remote accession Tak (from Japan) does not differ substantially from the Scandinavian accessions of subsp. *ruderalis*. The separation of the three taxa is also substantiated by the observation that each is associated with its own chloroplast and mitochondrial haplotype group. The present taxonomic treatment of *M. polymorpha ruderalis*, *M. polymorpha polymorpha* and *M. polymorpha montivagans* is intraspecific, as subspecies. This treatment has been questioned by Schuster (1992) and Damsholt (2002) arguing that the taxa are morphologically differentiated, having largely non-overlapping distribution areas. Kijak et al. (2018) came to the same conclusion based on data from two cpDNA regions. Whether to recognize the taxa at species or subspecies level depend to some extent on the species concept chosen and this will be discussed in another context. From a more practical point of view our study also raises a *memento* concerning phylogenetic inferences based on small sets of sequences. ILS and hybridization as well as differences in divergence among chromosomes, as evident from our study, may strongly affect the outcome of such analyses, especially for closely related species. Maybe, more so for bryophytes than for many other organisms if multiple hybridization events with transfer of small parts of the genome at a time is a widespread phenomenon (cf. Natcheva and Cronberg, 2007; Sawangproh et al., 2020).

## Conclusion

In conclusion, our data shows that the *Marchantia polymorpha*-complex comprises three recently diverged lineages, with relatively frequent hybridization and introgression, at least in a longer time perspective. Only limited parts of the genomes appear to be transferred between lineages at each occasion and one chromosome is less porous to gene transfer than the others. Alleles transferred between the genomes could still lead to improved adaptation, as they are immediately exposed to selection in the dominant haploid phase of the bryophyte life cycle.

## Data Availability Statement

The datasets generated for this study can be found at NCBI, https://www.ncbi.nlm.nih.gov under Bioproject PRJNA576577, and the raw data for *M. palacea* under Bioproject PRJNA362997.

## Author Contributions

UL and NC initiated the project. NC provided the plant material. UL and A-ML were responsible for DNA/RNA extractions. A-ML carried out the bioinformatics and statistics together with UL and WS, with input from NC. A-ML wrote the manuscript, in close collaboration with the other authors. PS provided chromosome assembly data and commented on the manuscript.

## Conflict of Interest

The authors declare that the research was conducted in the absence of any commercial or financial relationships that could be construed as a potential conflict of interest.

## Footnotes

1 https://jgi.doe.gov/data-and-tools/bbtools/

2 https://github.com/NBISweden/GAAS/

3 http://crsl4.github.io/PhyloNetworks.jl/latest/man/ticr_howtogetQuartetCFs/

## References

[B1] Anderson E. Hubricht L. (1938). Hybridization in Tradescantia. III. The evidence for introgressive hybridization. *Am. J. Bot.* 25 396–402. 10.1002/j.1537-2197.1938.tb09237.x

[B2] Atherton I. Bosanquet S. Lawley M. (2010). *Mosses and Liverworts of Britain and Ireland: A Field Guide.* London: British Bryological Society.

[B3] Bankevich A. Nurk S. Antipov D. Gurevich A. A. Dvorkin M. Kulikov A. S. (2012). SPAdes: a new genome assembly algorithm and its appications to single-cell sequencing. *J. Comput. Biol.* 19 455–477. 10.1089/cmb.2012.0021 22506599 PMC3342519

[B4] Bischler-Causse H. Boisselier-Dubayle M. C. (1991). Lectotypification of *Marchantia polymorpha* L. *J. Bryol.* 16 361–365.

[B5] Boisselier-Dubayle M. C. Bischler-Causse H. (1989). Electrophoretic studies in *Marchantia polymorpha* L. *J. Hattori Bot. Lab.* 67 297–311.

[B6] Boisselier-Dubayle M. C. Jubier M. F. Lejeune B. Bischler H. (1995). Genetic variability in the three subspecies of *Marchantia polymorpha* (Hepaticae): isozymes. RFLP and RAPD markers. *Taxon* 44 363–376. 10.2307/1223406

[B7] Bolger A. M. Lohse M. Usadel B. (2014). Trimmomatic: a flexible trimmer for Illumina Sequence Data. *Bioinformatics* 30 2114–2120. 10.1093/bioinformatics/btu170 24695404 PMC4103590

[B8] Bowman J. L. Kohchi T. Yamato K. T. Jenkins J. Shu S. Ishizaki K. (2017). Insights into Land Plant Evolution Garnered from the *Marchantia polymorpha* Genome. *Cell* 171:287–304.e15. 10.1016/j.cell.2017.09.030 28985561

[B9] Bradley R. K. Roberts A. Smoot M. Juvekar S. Do J. Dewey C. (2009). Fast statistical alignment. *PLoS Comput. Biol.* 5:e1000392. 10.1371/journal.pcbi.1000392 19478997 PMC2684580

[B10] Burgeff H. (1943). *Genetische Studien an Marchantia.* Jena: Gustav Fischer.

[B11] Capella-Gutiérrez S. Silla-Martínez J. M. Gabaldón T. (2009). trimAl: a tool for automated alignment trimming in large-scale phylogenetic analyses. *Bioinformatics* 25 1972–1973. 10.1093/bioinformatics/btp348 19505945 PMC2712344

[B12] Chin C.-S. Alexander D. H. Marks P. Klammer A. A. Drake J. Heiner C. (2013). Nonhybrid, finished microbial genome assemblies from long-read SMRT sequencing data. *Nat. Methods* 10 563–569. 10.1038/nmeth.2474 23644548

[B13] Damsholt K. (2002). *Illustrated Flora of Nordic Liverworts and Hornworts.* Lund: Nordic Bryological Society.

[B14] Dias-Alves T. Mairal J. Blum M. G. B. (2018). Loter: a software package to infer local ancestry for a wide range of species. *Mol. Biol. Evol.* 35 2318–2326. 10.1093/molbev/msy126 29931083 PMC6107063

[B15] Diop S. I. Subotic O. Giraldo-Fonseca A. Waller M. Kirbis A. Neubauer A. (2019). A pseudomolecule-scale genome assembly of the liverwort *Marchantia Polymorpha*. *Plant J.* 101 1378–1396. 10.1111/tpj.14602 31692190

[B16] Dowling T. E. Secor C. L. (1997). The role of hybridization and introgression in the diversification of animals. *Ann. Rev. Ecol. Syst.* 28 593–619. 10.1146/annurev.ecolsys.28.1.593

[B17] Durand E. Y. Patterson N. J. Reich D. Slatkin M. (2011). Testing for ancient admixture between closely related populations. *Mol. Biol. Evol.* 28 2239–2252. 10.1093/molbev/msr048 21325092 PMC3144383

[B18] Dutheil J. Y. Gaillard S. Stukenbrock E. H. (2014). MafFilter: a highly flexible and extensible multiple genome alignment files processor. *BMC Genomics* 15:53. 10.1186/1471-2164-15-53 24447531 PMC3904536

[B19] Edgar R. C. (2004). MUSCLE: multiple sequence alignment with high accuracy and high throughput. *Nucleic Acids Res.* 32 1792–1797. 10.1093/nar/gkh340 15034147 PMC390337

[B20] Fontaine M. C. Pease J. B. Steele A. Waterhouse R. M. Neafsey D. E. Sharakhov I. V. (2015). Extensive introgression in a malaria vector species complex revealed by phylogenomics. *Science* 347 1258524–1258524. 10.1126/science.1258524 25431491 PMC4380269

[B21] Galtier N. Daubin V. (2008). Dealing with incongruence in phylogenomic. *Philos. Trans. R. Soc. Lond. B Biol. Sci.* 363 4023–4029. 10.1098/rstb.2008.0144 18852109 PMC2607408

[B22] Garrison E. Marth G. (2012). Haplotype-based variant detection from short-read sequencing. *arXiv [Preprint].* Available online at: arXiv:1207.3907v2 (accessed 1 August 2019).

[B23] Gogarten J. P. Townsend J. P. (2005). Horizontal gene transfer, genome innovation and evolution. *Nat. Rev. Microbiol.* 3 679–687. 10.1038/nrmicro1204 16138096

[B24] Grabherr M. G. Haas B. J. Yassour M. Levin J. Z. Thompson D. A. Amit I. (2011). Full-length transcriptome assembly from RNA-seq data without a reference genome. *Nat. Biotechnol.* 29 644–652. 10.1038/nbt.1883 21572440 PMC3571712

[B25] Grabherr M. G. Russell P. Meyer M. Mauceli E. Alföldi J. Palma F. D. (2010). Genome-wide synteny through highly sensitive sequence alignment: Satsuma. *Bioinformatics* 26 1145–1151. 10.1093/bioinformatics/btq102 20208069 PMC2859124

[B26] Grant P. R. Grant B. R. Petren K. (2005). Hybridization in the recent past. *Am. Nat.* 166 56–67. 10.1086/430331 15937789

[B27] Gurevich A. Saveliev V. Vyahhi N. Tesler G. (2013). QUAST: quality assessment tool for genome assemblies. *Bioinformatics* 29 1072–1075. 10.1093/bioinformatics/btt086 23422339 PMC3624806

[B28] Harrison R. G. Larson E. L. (2014). Hybridization, introgression, and the nature of species boundaries. *J. Heredity* 105 795–809. 10.1093/jhered/esu033 25149255

[B29] Healey A. Furtado A. Cooper T. Henry R. J. (2014). Protocol: a simple method for extracting next-generation sequencing quality genomic DNA from recalcitrant plant species. *Plant Methods* 10:21. 10.1186/1746-4811-10-21 25053969 PMC4105509

[B30] Heliconius Genome Consortium. (2012). Butterfly genome reveals promiscuous exchange of mimicry adaptations among species. *Nature* 487 94–98. 10.1038/nature11041 22722851 PMC3398145

[B31] Hickey G. Paten B. Earl D. Zerbino D. Haussler D. (2013). HAL: a hierarchical format for storing and analyzing multiple genome alignments. *Bioinformatics* 29 1341–1342. 10.1093/bioinformatics/btt128 23505295 PMC3654707

[B32] Holt C. Yandell M. (2011). MAKER2: an annotation pipeline and genome-database management tool for second-generation genome projects. *BMC Bioinformatics* 12:491. 10.1186/1471-2105-12-491 22192575 PMC3280279

[B33] Huelsenbeck J. P. Ronquist F. (2001). MRBAYES: Bayesian inference of phylogenetic trees. *Bioinformatics* 17 754–755. 10.1093/bioinformatics/17.8.754 11524383

[B34] Kahle D. Wickham H. (2013). ggmap: Spatial visualization with ggplot2. *R J.* 5 144–161.

[B35] Keane T. M. Creevey C. J. Pentony M. M. Naughton T. J. Mclnerney J. O. (2006). Assessment of methods for amino acid matrix selection and their use on empirical data shows that ad hoc assumptions for choice of matrix are not justified. *BMC Evol. Biol.* 6:29. 10.1186/1471-2148-6-29 16563161 PMC1435933

[B36] Kijak H. Łodyga W. Odrzykoski I. J. (2018). Sequence diversity of two chloroplast genes: rps4 and tRNAGly (UCC), in the liverwort *Marchantia polymorpha*, an emerging plant model system. *Acta Soc. Bot. Pol.* 87:3573.

[B37] Kim D. Langmead B. Salzberg S. L. (2015). HISAT: a fast spliced aligner with low memory requirements. *Nat. Methods* 12 357–360. 10.1038/nmeth.3317 25751142 PMC4655817

[B38] Larget B. R. Kotha S. K. Dewey C. N. Ané C. (2010). BUCKy: gene tree/species tree reconciliation with Bayesian concordance analysis. *Bioinformatics* 26 2910–2911. 10.1093/bioinformatics/btq539 20861028

[B39] Li H. Handsaker B. Wysoker A. Fennell T. Ruan J. Homer N. (2009). Genome Project Data Processing Subgroup. 2009. The Sequence alignment/map (SAM) format and SAMtools. *Bioinformatics* 25 2078–2079. 10.1093/bioinformatics/btp352 19505943 PMC2723002

[B40] Luo R. Liu B. Xie Y. Li Z. Huang W. Yuan J. (2012). SOAPdenovo2: an empirically improved memory-efficient short-read de novo assembler. *Gigascience* 1:18. 10.1186/2047-217X-1-18 23587118 PMC3626529

[B41] Mallet J. (2005). Hybridization as an invasion of the genome. *Trends Ecol. Evol.* 20 229–237. 10.1016/j.tree.2005.02.010 16701374

[B42] Mallet J. (2007). Hybrid speciation. *Nature* 446 279–283. 17361174 10.1038/nature05706

[B43] Martin S. H. Dasmahapatra K. K. Nadeau N. J. Salazar C. Walters J. R. Simpson F. (2013). Genome-wide evidence for speciation with gene flow in Heliconius butterflies. *Genome Res.* 23 1817–1828. 24045163 10.1101/gr.159426.113 PMC3814882

[B44] Martin S. H. Jiggins C. D. (2017). Interpreting the genomic landscape of introgression. *Curr. Opin. Genet. Dev.* 47 69–74. 10.1016/j.gde.2017.08.007 28923541

[B45] Mirarab S. Reaz R. Bayzid M. S. Zimmermann T. Swenson M. S. Warnow T. (2014). ASTRAL: genome-scale coalescent-based species tree estimation. *Bioinformatics* 30 i541–i548. 10.1093/bioinformatics/btu462 25161245 PMC4147915

[B46] Mirarab S. Warnow T. (2015). ASTRAL-II: coalescent-based species tree estimation with many hundreds of taxa and thousands of genes. *Bioinformatics* 31 i44–i52. 10.1093/bioinformatics/btv234 26072508 PMC4765870

[B47] Mossel E. Roch S. (2010). Incomplete lineage sorting: consistent phylogeny estimation from multiple loci. *IEEE/ACM Trans. Comput. Biol. Bioinform.* 7 166–171. 10.1109/tcbb.2008.66 20150678

[B48] Narasimhan V. Danecek P. Scally A. Xue Y. Tyler-Smith C. Durbin R. (2016). BCFtools/RoH: a hidden Markov model approach for detecting autozygosity from next-generation sequencing data. *Bioinformatics* 32 1749–1751. 10.1093/bioinformatics/btw044 26826718 PMC4892413

[B49] Natcheva R. Cronberg N. (2004). What do we know about hybridization among bryophytes in nature? *Can. J. Bot.* 82 1687–1704. 10.1139/b04-139

[B50] Natcheva R. Cronberg N. (2007). Maternal transmission of cytoplasmic DNA in interspecific hybrids of peat mosses. *Sphagnum (Bryophyta)*. *J. Evol. Biol.* 20 1613–1616. 10.1111/j.1420-9101.2007.01341.x 17584253

[B51] Nees C. G. (1838). *Naturgeschichte der Europäischen Lebermoose mit besonderer Beziehung auf Schlesien und die Oertlichebkeiten des Riesengebirgs*, Vol. 4 Breslau: Grass, Barth & Co.

[B52] Nishiyama T. Wolf P. G. Kugita M. Sinclair R. B. Sugita M. Sugiura C. (2004). Chloroplast phylogeny indicates that bryophytes are monophyletic. *Mol. Biol. Evol.* 21 1813–1819. 10.1093/molbev/msh203 15240838

[B53] O’Hanlon M. E. (1926). Germination of spores and early stages in development of gametophyte of *Marchantia polymorpha*. *Bot. Gazette* 82 215–222. 10.1086/333650

[B54] Paradis E. Schliep K. (2019). Ape5.0: an environment for modern phylogenetics and evolutionary analyses in R. *Bioinformatics* 35 526–528. 10.1093/bioinformatics/bty633 30016406

[B55] Parra G. Bradnam K. Korf I. (2007). CEGMA: a pipeline to accurately annotate core genes in eukaryotic genomes. *Bioinformatics* 23 1061–1067. 10.1093/bioinformatics/btm071 17332020

[B56] Paten B. Diekhans M. Earl D. John J. Ma J. Suh B. (2011a). Cactus graphs for genome comparisons. *J. Comput. Biol.* 18 469–481. 10.1089/cmb.2010.0252 21385048 PMC8884192

[B57] Paten B. Earl D. Nguyen N. Diekhans M. Zerbino D. Haussler D. (2011b). Cactus: algorithms for genome multiple sequence alignment. *Genome Res.* 21 1512–1528. 10.1101/gr.123356.111 21665927 PMC3166836

[B58] Paton J. A. (1999). *The Liverwort Flora of the British Isles.* Colchester: Harley Books.

[B59] Payseur B. A. Rieseberg L. H. (2016). A genomic perspective on hybridization and speciation. *Mol. Ecol.* 25 2337–2360. 10.1111/mec.13557 26836441 PMC4915564

[B60] Pertea M. Pertea G. M. Antonescu C. M. Chang T. C. Mendell J. T. Salzberg S. L. (2015). StringTie enables improved reconstruction of a transcriptome from RNA-seq reads. *Nat. Biotechnol.* 33 290–295. 10.1038/nbt.3122 25690850 PMC4643835

[B61] Pfeifer B. Kapan D. D. (2017). Estimates of introgression as a function of pairwise distances. *bioRxiv [Preprint]* 10.1101/154377 PMC6480520 31014244

[B62] Pfeifer B. Wittelsbürger U. Ramos-Onsins S. E. Lercher M. J. (2014). PopGenome: an efficient Swiss army knife for population genomic analyses in R. *Mol. Biol. Evol.* 31 1929–1936. 10.1093/molbev/msu136 24739305 PMC4069620

[B63] Qiu Y.-L. Li L. Wang B. Chen Z. Knoop V. Groth-Malonek M. (2006). The deepest divergences in land plants inferred from phylogenomic evidence. *Proc. Natl. Acad. Sci. U.S.A.* 103 15511–15516. 10.1073/pnas.0603335103 17030812 PMC1622854

[B64] Radhakrishnan G. (2017). *Tracing the Evolution of the Arbuscular Mycorrhizal Symbiosis in the Plant Lineage.* Doctoral thesis, University of East Anglia, Norwich.

[B65] Rieseberg L. H. Archer M. A. Wayne R. K. (1999). Transgressive segregation, adaptation and speciation. *Heredity* 83 363–372. 10.1038/sj.hdy.6886170 10583537

[B66] Rieseberg L. H. Raymond O. Rosenthal D. M. Lai Z. Livingstone K. Nakazato T. (2003). Major ecological transitions in wild sunflowers facilitated by hybridization. *Science* 301 1211–1216. 10.1126/science.1086949 12907807

[B67] Roberts R. J. Carneiro M. O. Schatz M. (2013). The advantages of SMRT sequening. *Genome Biol.* 14:405. 10.1186/gb-2013-14-7-405 PMC3953343 23822731

[B68] Ronquist F. Huelsenbeck J. P. (2003). MrBayes 3: bayesian phylogenetic inference under mixed models. *Bioinformatics* 19 1572–1574. 10.1093/bioinformatics/btg180 12912839

[B69] Ronquist F. Teslenko M. van der Mark P. Ayres D. L. Darling A. Höhna S. (2012). MrBayes 3.2: efficient Bayesian phylogenetic inference and model choice across a large model space. *Syst. Biol.* 61 539–542. 10.1093/sysbio/sys029 22357727 PMC3329765

[B70] Sawangproh W. (2019). *Gene transfer by interspecific hybridization in bryophytes.* Lund: Media-Tryck, Lund University, Sweden.

[B71] Sawangproh W. Hedenäs L. Lang A. S. Hansson B. Cronberg N. (2020). Gene transfer across species boundaries in bryophytes: evidence from major life cycle stages in Homalothecium lutescens and *H. sericeum*. *Ann. Bot.* 125 565–579. 10.1093/aob/mcz209 31872857 PMC7102947

[B72] Schuster R. M. (1983). “Phytogeography of the Bryophyta,” in *New Manual of Bryology 1*, ed. Schuster R. M. (Nichinan: Hattori Botanical Laboratory), 463–626.

[B73] Schuster R. M. (1992). *The Hepaticae and Anthocerotae of North America.* New York, NY: Columbia University Press.

[B74] Seehausen O. (2004). Hybridization and adaptive radiation. *Trends Ecol. Evol.* 19 198–207. 10.1016/j.tree.2004.01.003 16701254

[B75] Shaw A. J. (1994). Systematics of Mielichhoferia (Bryaceae: Musci). III. Hybridization between *M. elongata* and *M. mielichhoferiana*. *Am. J. Bot.* 81 782–790. 10.1002/j.1537-2197.1994.tb15515.x

[B76] Shaw A. J. (1998). “Genetic analysis of a hybrid zone in Mielichhoferia (Musci),” in *Bryology for the Twenty-First*, eds Bates J. W. Ashton N. W. Duckett J. G. (Leeds: Century), 161–174. 10.1201/9781315138626-12

[B77] Shen W. Le S. Li Y. Hu F. (2016). SeqKit: a cross-platform and Ultrafast Toolkit for FASTA/Q File Manipulation. *PLoS One* 11:e0163962. 10.1371/journal.pone.0163962 27706213 PMC5051824

[B78] Shimamura M. (2016). *Marchantia polymorpha*: taxonomy, phylogeny and morphology of a model system. *Plant Cell Physiol.* 57 230–256. 10.1093/pcp/pcv192 26657892

[B79] Simão F. A. Waterhouse R. M. Ioannidis P. Kriventseva E. V. Zdobnov E. M. (2015). BUSCO: assessing genome assembly and annotation completeness with single-copy orthologs. *Bioinformatics* 31 3210–3212. 10.1093/bioinformatics/btv351 26059717

[B80] Smit A. F. A. Hubley R. (2008-2015). *RepeatModeler Open-1.0.* Availale online at: http://www.repeatmasker.org

[B81] Smit A. F. A. Hubley R. Grenn P. (2013-2015). *RepeatMasker Open-4.0*. Availale online at: http://www.repeatmasker.org

[B82] Soltis P. S. Soltis D. E. (2009). The role of hybridization in plant speciation. *Annu. Rev. Plant Biol.* 60 561–588. 10.1146/annurev.arplant.043008.092039 19575590

[B83] Stamatakis A. (2014). RAxML version 8: a tool for phylogenetic analysis and post-analysis of large phylogenies. *Bioinformatics* 30 1312–1313. 10.1093/bioinformatics/btu033 24451623 PMC3998144

[B84] Stanke M. Morgenstern B. (2005). AUGUSTUS: a web server for gene prediction in eukaryotes that allows user-defined constraints. *Nucleic Acids Res.* 33 W465–W467. 15980513 10.1093/nar/gki458 PMC1160219

[B85] Trapnell C. Hendrickson D. G. Sauvageau M. Goff L. Rinn J. L. Pachter L. (2013). Differential analysis of gene regulation at transcript resolution with RNA-seq. *Nat. Biotechnol.* 31 46–53. 10.1038/nbt.2450 23222703 PMC3869392

[B86] Villarreal A. J. C. Crandall-Stotler B. J. Hart M. L. Long D. G. Forrest L. L. (2016). Divergence times and the evolution of morphological complexity in an early land plant lineage (Marchantiopsida) with a slow molecular rate. *New Phytol.* 209 1734–1746. 10.1111/nph.13716 26505145

[B87] Wang Y. Coleman-Derr D. Chen G. Gu Y. Q. (2015). OrthoVenn: a web server for genome wide comparison and annotation of orthologous clusters across multiple species. *Nucleic Acids Res.* 43 W78–W84. 10.1093/nar/gkv487 25964301 PMC4489293

[B88] Wang Y. Tang H. Debarry J. D. Tan X. Li J. Wang X. (2012). MCScanX: a toolkit for detection and evolutionary analysis of gene synteny and collinearity. *Nucleic Acids Res.* 40:e49. 10.1093/nar/gkr1293 22217600 PMC3326336

[B89] Ward B. J. van Oosterhout C. (2016). HYBRIDCHECK?: software for the rapid detection, visualization and dating of recombinant regions in genome sequence data. *Mol. Ecol. Resour.* 16 534–539. 10.1111/1755-0998.12469 26394708

[B90] Wickett N. J. Mirarab S. Nguyen N. Warnow T. Carpenter E. Matasci N. (2014). Phylotranscriptomic analysis of the origin and early diversification of land plants. *Proc. Natl. Acad. Sci. U.S.A.* 111 E4859–E4868. 25355905 10.1073/pnas.1323926111 PMC4234587

