# Supplementary figures and images for: Evolutionary History of the Marchantia polymorpha Complex
Source: Front Plant Sci. 2020 Jun 26;11:829. doi: 10.3389/fpls.2020.00829 (PMC7332582; doi:10.3389/fpls.2020.00829)

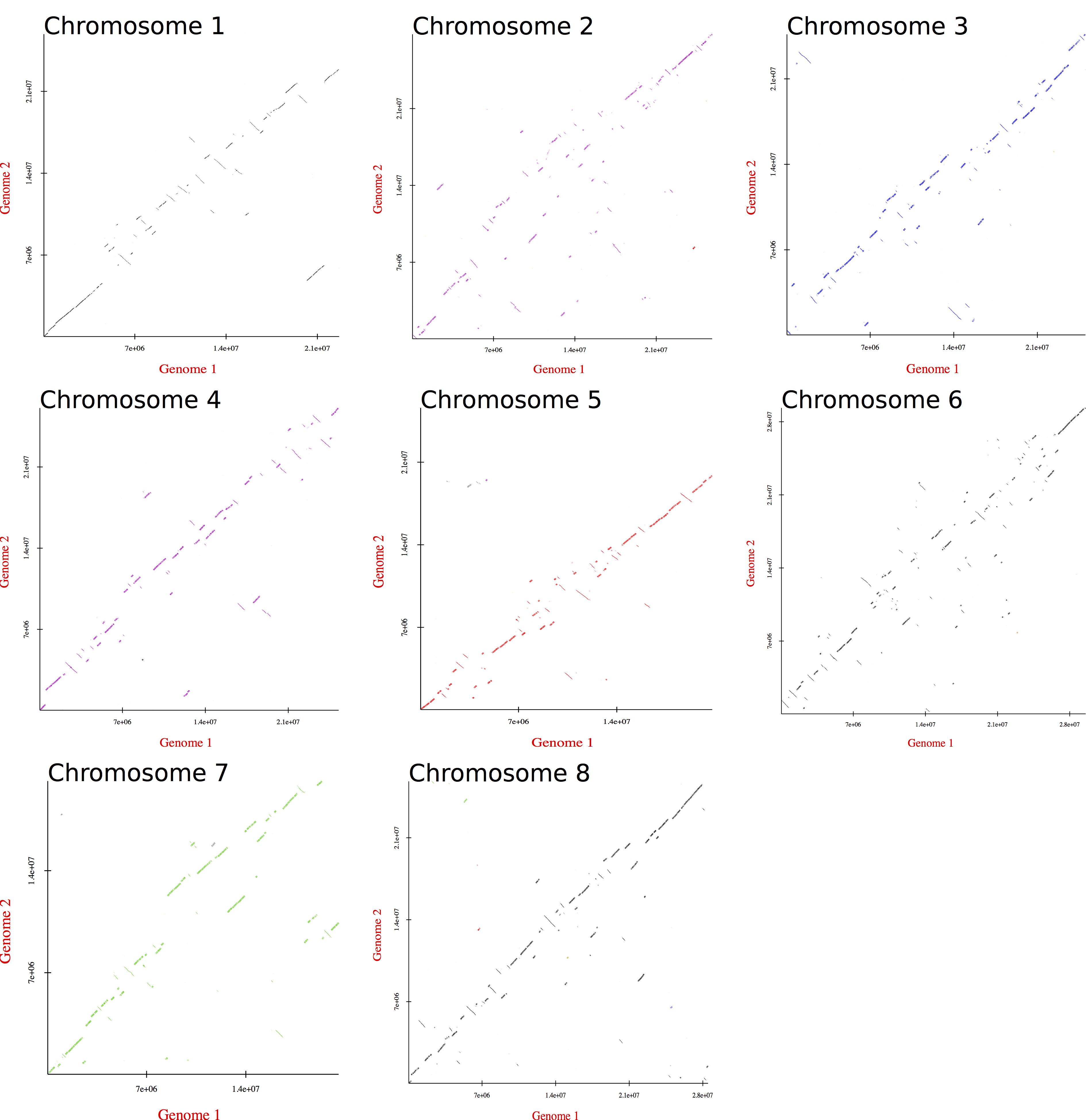

Supplement: Supplementary file 1 [file Image_1.jpg]

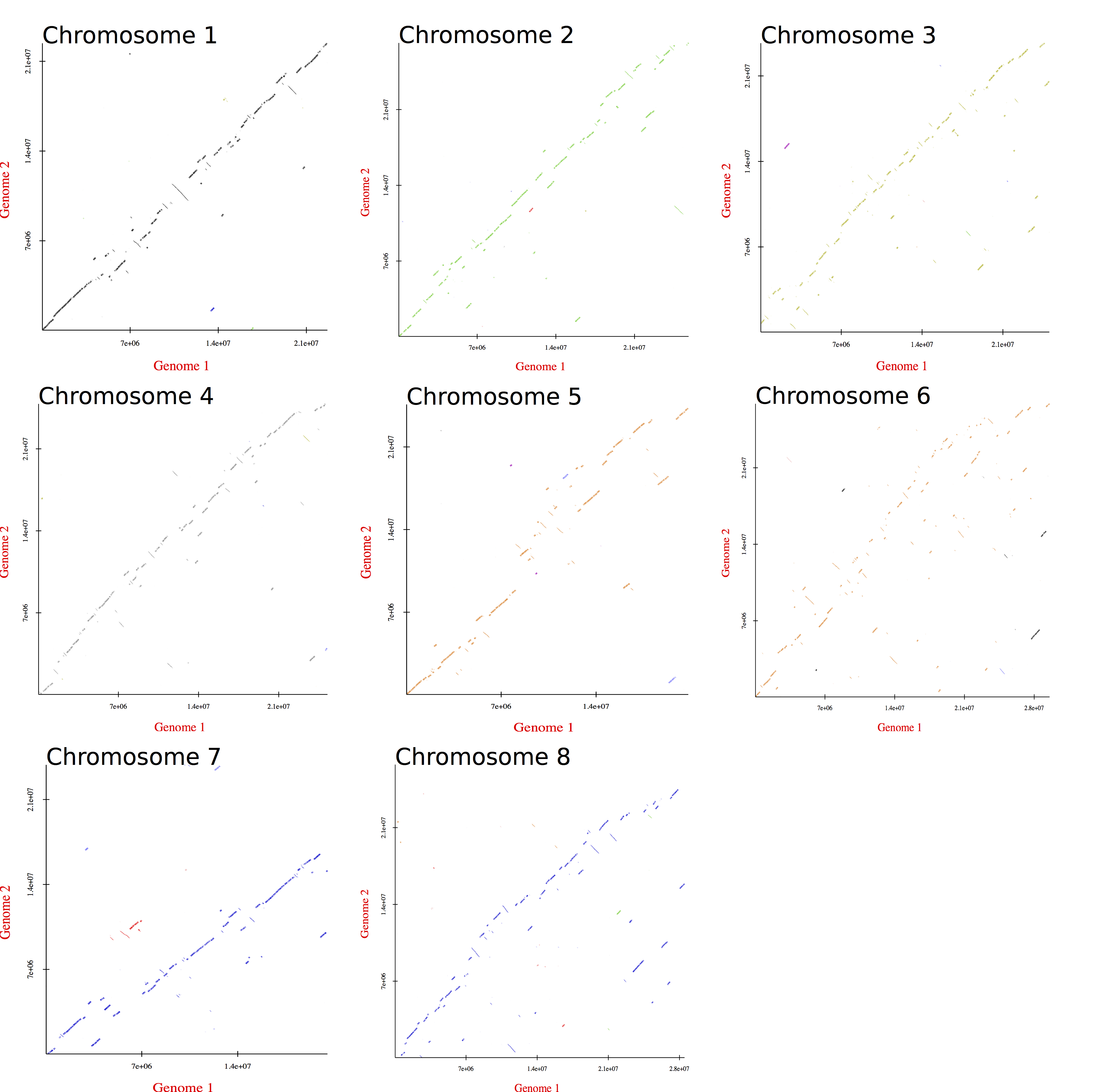

Supplement: Supplementary file 2 [file Image_2.jpg]

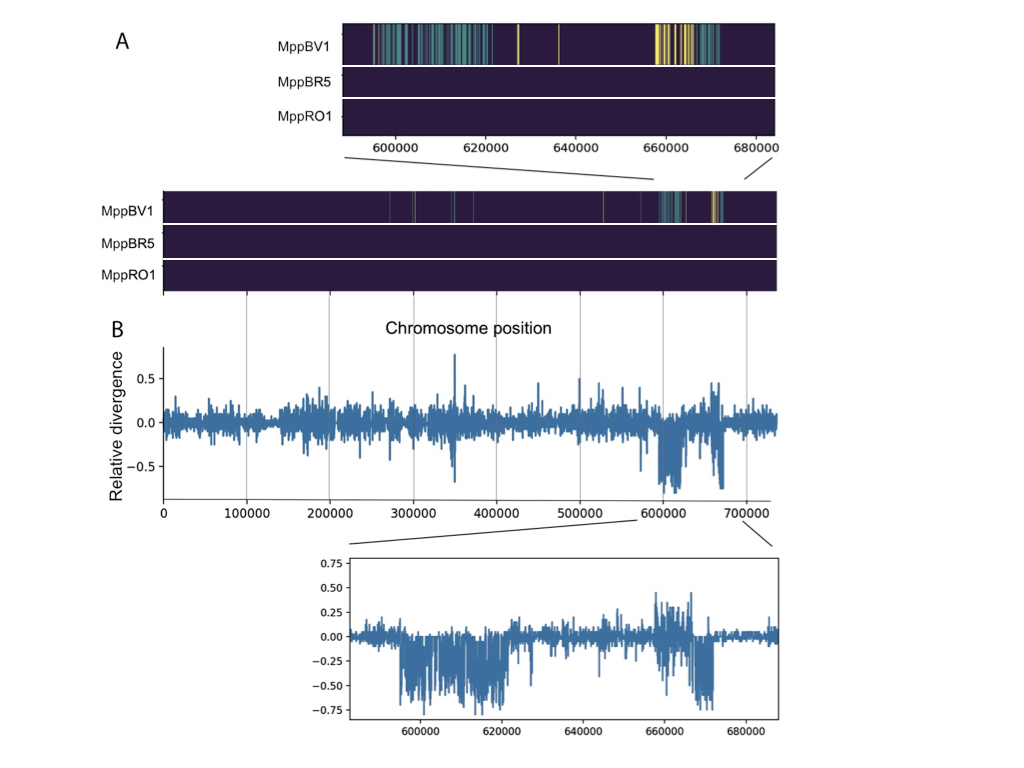

Supplement: Supplementary file 3 [file Image_3.jpeg]
