# Supplementary material for: Evolutionary History of the Marchantia polymorpha Complex
Source: Front Plant Sci. 2020 Jun 26;11:829. doi: 10.3389/fpls.2020.00829 (PMC7332582; doi:10.3389/fpls.2020.00829)
Supplement: Supplementary file 4 [file Data_Sheet_1.docx]

Supplementary Material

Supplemental Table 1. The sample ID, localities, geographic coordinates, habitats and date of sampling of *Marchantia polymorpha* plants. All DNA studied samples supplied from the living samples that were cultured and maintained in the growth chamber room at Dept. of Biology, Lund University (Sweden).

| **Sample ID** | **Locality** | **Geographic coordinates** | **Habitat** | **Date of sampling** | **Remark** |
| --- | --- | --- | --- | --- | --- |
| ***M. polymorpha ruderalis*** | |  |  |  |  |
| **Tak-1 + Tak-2** |  |  |  |  |  |
| **MprBU7** | The vicinity of Ogoya village, Raven's combe (Sofia district, Bulgaria) | 42°54'42.71"N 23°30'49.06"E | Swampy area along a rivulet, c. 800 m asl | 31 March 2012 | Only female thalli |
| **MprBD3** | Bydalen in Jämtland (Sweden) | 62°01'59.03"N  12°23'41.60"E | Parking place by a mountain cottage | 2013-08-11 | Unknown gender |
| **MprSA8** | Murjek, east of the railway | 66°28'55.91"N  20°52'59.76"E | Wet land | in 2012 | Unknown gender |
| **MprLY4** | Lyngsjö in Skåne Province (Sweden) | 55°55'53.05"N  14°04'04.14"E | Lake shore by the old bathing place | 2012-10-23 | Only female thalli found |
| **MprRÖ2** | Röan in Skåne Province (Sweden) | 55°59'35.93"N  13°30'45.32"E | Moist pasture, 340 m southwest of Röans gård | 2015-09-25 | Unknown gender |
| ***M. polymorpha montivagans*** | |  |  |  |  |
| **MpmSA2** | Sarvesvagge | 67°15'11.72"N  17°39'26.82"E | Sloping fen in birch forest | in 2012 |  |
| **MpmBV2** | Bredvalla in Dalarna (Sweden) | 61°05'01.53"N 13°08'05.63"E | Verge of small stream | 25 September 2015 | Unknown gender |
| **MpmBU3** | The vicinity of Ogoya village, Raven's combe (Sofia district, Bulgaria) | 42°54'42.71"N 23°30'49.06"E | Swampy area along a rivulet, c. 800 m asl | 31 March 2012 | Only female thalli found |
| ***M. polymorpha polymorpha*** | |  |  |  |  |
| **MppBR5** | Lönhults sommarby near Brösarp in Skåne Province (Sweden) | 55°43'49.25"N 14°02'38.63"E | Moist pasture | 2012-06-09 | Only male thalli found |
| **MppRÖ3** | Röan in Skåne Province (Sweden) | 55°59'35.93"N  13°30'45.32"E | Moist pasture | 2015 | Unknown gender |
| **MppBV1** | Bredvalla in Dalarna (Sweden) | 61°05'01.53"N 13°08'05.63"E | Verge of small stream | 25 September 2015 | Unknown gender |

Supplemental Table 2. Assembly statistics and completeness for the assemblies and annotations used in this study. MPM = *Marchantia polymorpha* subsp. *montivagans*, MPP = *M. polymorpha* subsp. *polymorpha*, MPR = *M. polymorpha* subsp. *ruderalis*, MPA = *M. paleacea*.

|  | **MPM** | **MPP** | **MPR** | **MPA** |
| --- | --- | --- | --- | --- |
| **# contigs (>= 1000 bp)** | 2585 | 2600 | 2957 | 9482 |
| **Total length (>= 1000 bp)** | 225663078 | 222577407 | 225761139 | 232005299 |
| **Largest contig size** | 2032864 | 2164001 | 6192411 | 532063 |
| **N50** | 589422 | 368251 | 1366373 | 77560 |
| **NG50** | 417213 | 269767 | 1023827 | 63181 |
| **LG50** | 173 | 262 | 77 | 1217 |
| **Assembly completeness (CEGMA/BUSCO)** | 91.94%/89.7% | 91.53%/88.5% | 92.74%/89.7% | 90.73%/88.8% |
| **Predicted repeat content in assembly** | 34.82 | 34.62 | 27.89 | 39.79 % |
| **Annotated gene models** | 18806 | 17374 | 19138 | 14478 |
| **Annotation completeness (BUSCO % complete/fragmented/missing)** | 92.8/5/2.2 | 86.8/9.9/3.3 | 97.4/0.3/2.3 | 90.8/5.9/3.3 |

**Supplemental Figure 1**. Synteny dot plot where "Genome 1" represent subsp. *ruderalis*chromosomes and "Genome 2" represent subsp. *montivagans*pseudochromosomes.

**Supplemental Figure 2**. Synteny dot plot where "Genome 1" represent subsp.  *ruderalis*chromosomes and "Genome 2" represent subsp.  *polymorpha* pseudochromosomes.

**Supplemental Figure 3**. Evidence for limited introgression is observed for one subsp. *polymorpha* individual. **(A)** Local ancestry inference for three subsp. *polymorpha* individuals derived from Loter software. Image plot of pseudo-chromosome 1 illustrating ancestral origin, subsp. *montivagans* (green), *ruderalis* (yellow) and *polymorpha* (purple). The x-axis shows SNP number along chromosome 1. **(B)** Plot of difference in genetic diversity between on the one hand MppBV1 versus subsp. *montivagans* individuals, and on the other hand MpmBV1 versus other subsp. *polymorpha* individuals (d_MpmBV1__*_montivagans_* – d_MpmBV1__*_polymorpha_*).
